# Altered Plasma Metabolic Profiles in Chinese Patients With Multiple Sclerosis

**DOI:** 10.3389/fimmu.2021.792711

**Published:** 2021-12-15

**Authors:** Fan Yang, Shao-chang Wu, Zong-xin Ling, Shan Chao, Li-juan Zhang, Xiu-mei Yan, Lin He, Li-mei Yu, Long-you Zhao

**Affiliations:** ^1^ Key Laboratory of Cell Engineering in Guizhou Province, Affiliated Hospital of Zunyi Medical University, Zunyi, China; ^2^ Bio-X Institutes, Key Laboratory for the Genetics of Developmental and Neuropsychiatric Disorders (Ministry of Education), Shanghai Jiao Tong University, Shanghai, China; ^3^ Institutes for Shanghai Pudong Decoding Life, Research Center for Lin He Academician New Medicine, Shanghai, China; ^4^ Department of Geriatrics and Clinical Laboratory, Lishui Second People’s Hospital, Lishui, China; ^5^ Collaborative Innovation Center for Diagnosis and Treatment of Infectious Diseases, State Key Laboratory for Diagnosis and Treatment of Infectious Diseases, National Clinical Research Center for Infectious Diseases, The First Affiliated Hospital, School of Medicine, Zhejiang University, Hangzhou, China; ^6^ Institute of Microbe & Host Health, Linyi University, Linyi, China

**Keywords:** multiple sclerosis, differentially abundant metabolite, cytokine, chemokine, peripheral immunoinflammatory response, disease course

## Abstract

Multiple sclerosis (MS) is an autoimmune disease that leads to the demyelination of nerve axons. An increasing number of studies suggest that patients with MS exhibit altered metabolic profiles, which might contribute to the course of MS. However, the alteration of metabolic profiles in Chinese patients with MS and their potential roles in regulating the immune system remain elusive. In this study, we performed a global untargeted metabolomics approach in plasma samples from 22 MS-affected Chinese patients and 21 healthy subjects. A total of 42 differentially abundant metabolites (DAMs) belonging to amino acids, lipids, and carbohydrates were identified in the plasma of MS patients and compared with those in healthy controls. We observed an evident reduction in the levels of amino acids, such as L-tyrosine, L-isoleucine, and L-tryptophan, whereas there was a great increase in the levels of L-glutamic acid and L-valine in MS-affected patients. The levels of lipid and carbohydrate metabolites, such as sphingosine 1-phosphate and myo-inositol, were also reduced in patients with MS. In addition, the concentrations of proinflammatory cytokines, such as IL-17 and TNF-α, were significantly increased, whereas those of several anti-inflammatory cytokines and chemokines, such as IL-1ra, IL-7, and MIP-1α, were distinctly reduced in the plasma of MS patients compared with those in healthy subjects. Interestingly, some DAMs, such as L-tryptophan and sphingosine 1-phosphate, showed an evident negative correlation with changes in the level of TNF-α and IL-17, while tightly positively correlating with altered concentrations of anti-inflammatory cytokines and chemokines, such as MIP-1α and RANTES. Our results revealed that altered metabolomic profiles might contribute to the pathogenesis and course of MS disease by modulating immuno-inflammatory responses in the peripheral system, which is essential for eliciting autoimmune responses in the central nervous system, thus resulting in the progression of MS. This study provides potential clues for developing therapeutic strategies for MS in the near future.

## 1 Introduction

Multiple sclerosis (MS), an immunoinflammatory disease, is caused by the chronic demyelination of nerve axons and, thus, disrupts the function of the central nervous system (CNS), eventually leading to severe disability, such as autonomic dysfunction, paralysis, disability of motor control, and cognitive impairment of the brain (1–3). According to the latest open-source data of MS global epidemiology derived from the Atlas of MS (www.atlasofms.org), which is compiled and updated by the Multiple Sclerosis International Federation (MSIF), it is estimated that 2.8 million people are affected by MS worldwide. The prevalence has risen to 35.9 per 100 000 people since 2013 (4), and women are twice as likely to be affected (4). Nevertheless, to date, no comprehensive statistical data on MS epidemiology have been reported in China, even though multiple regional studies have revealed the incidence of MS in the Chinese population (5–9). Phenotypically, the vast majority of patients begin with a clinically isolated syndrome (CIS) before developing definite MS. Patients who have experienced at least two relapses are described as suffering from relapsing-remitting multiple sclerosis (RRMS), while approximately 15% of patients enter the progressive phase upon the onset of the disease, which is termed primary progressive multiple sclerosis (PPMS) (10). The causal factors of MS include genetic and epigenetic cues as well as environmental elements (11), such as low intake of vitamin D, diet, and smoking (12–14). Studies have verified that multiple genetic variants confer an MS risk through the regulation of immune responses (15–17).

Currently, metabolomics is an effective solution for identifying the predictive biomarkers and uncovering the pathogenesis of diseases (18, 19). In recent decades, numerous metabolomic studies that identify differentially abundant metabolites associated with MS symptoms in MS-affected patients (20–46), and in animal models of MS, experimental autoimmune encephalomyelitis (EAE) (47–49), have been performed. Nevertheless, metabolomic investigations on Chinese patients with MS have been very rare. The interaction between metabolic pathways and the immune system has been identified to play a vital role in modulating autoimmunity (50–53), promoting the pathogenesis of several diseases, such as various cancers and Alzheimer’s disease (54, 55). A previous study suggested that sphingolipid lactosylceramide boosted inflammation and neurodegeneration in an EAE model (56). Further investigations revealed that sphingolipid metabolism in astrocytes stimulated inflammation within the CNS *via* the trigger mechanisms of cPLA2-MAVS protein interaction-enhanced NF-κB signaling (57). Multiple studies have also emphasized the indispensable role of sphingolipid metabolites in modulating inflammatory diseases (58–64). In addition, sustained intake of short-chain fatty acids (SCFAs) like propionic acid was reported to significantly increase the population of regulatory T (Treg) cells in MS patients. In contrast, it significantly decreased the populations of T helper type 1 (Th1) and type 17 (Th17) cells (65), which produce proinflammatory cytokines and mediate inflammation in the CNS mainly in patients with MS (66) as well as in those with EAE (67–71). Studies in animal models of MS and other diseases have also demonstrated that SCFAs, such as propionic acid and butyrate, facilitate the generation of gut-related Treg cells and alleviate immune responses, thus resulting in disease remission (72–78). Moreover, amino acids, such as tryptophan metabolites, have been shown to function as a key class of immunomodulatory mediators capable of regulating immune homeostasis in MS (79–83). The indole-based compounds produced by the metabolism of tryptophan by gut microbiota can interact with and activate the transcription factor aryl hydrocarbon receptor (AHR), thereby regulating the transcription program and modulating autoimmune reactions (24, 84–90). A series of studies has also shown that metabolites of dietary tryptophan produced by gut flora regulate the immunoinflammatory responses of CNS in the EAE model *via* AHR-mediated signaling (91–93).

The mechanisms of the metabolic-immune crosstalk regulating the MS disease, particularly in Caucasian populations and in animal models of MS, have been well illustrated in numerous studies. However, little is known about the characteristics of the peripheral metabolic profiles in Chinese patients with stable MS, as well as their role in modulating immune responses that control the course of the disease. In this study, we revealed a significantly altered metabolic profile and immune responses in the peripheral system of Chinese patients with MS, in which metabolites of amino acids and sphingolipids might not only modulate peripheral proinflammatory responses but also drive the inflammatory process within the CNS. These findings outlined the presence of an immunometabolic network that controls the immune responses of the peripheral system in Chinese patients with MS. This network potentially maintains the disease course and shapes inflammation in the CNS, which is essential for initiating and dominating the progressive phase of MS.

## 2 Materials and Methods

### 2.1 Subject Recruitment

In total, 22 Chinese patients with MS, including 20 RRMS and two PPMS cases, who were diagnosed based on the revised version of the 2005 McDonald criteria (94), were recruited from eastern China from December 2018 to January 2020. Meanwhile, 21 sex- and age-matched healthy volunteers were also enrolled as controls group (**Table 1**). Informed consent was acquired from each participant prior to the recruitment. The experimental protocols of this study were approved by the Ethics Committee of the Second People’s Hospital of Lishui (Zhejiang, China). None of the enrolled patients had received treatment of beta-interferon, steroids, or other immunosuppressive drugs in the three months before participating in the study. Exclusion criteria were as follows: age less than 20 years; body mass index (BMI) greater than 30.0; pregnancy; chronic diseases such as hyperlipidemia, hypertension, or diabetes mellitus; known microbial (bacterial or fungal) or virus infections; and other autoimmune diseases.

**Table 1:** Characteristics of patients with MS and healthy subjects.

Characteristics	Patients with MS	Healthy controls	*P* value
Number	22	21	NA
Sex, male/female	8/14	8/13	NA
Age (y), mean ± SD.	34.8 ± 7.5	33.3 ± 8.5	NS
BMI (kg/m^2^), mean ± SD.	21.6 ± 2.6	22.1 ± 3.6	NS
Duration (y), mean ± SD.	7.4 ± 5.0	NA	NA
MS subtype, RRMS	20	0	
MS subtype, PPMS	2	0	
Hypertension	0	0	
Hyperlipidemia	0	0	
Diabetes mellitus	0	0	
Autoimmune diseases	0	0	
Active infections	0	0	
Immunosuppressive medications within 3 months	0	0	

NA, not available; SD, standard deviation; NS, not significant; BMI, body mass index.

### 2.2 Plasma Sample Collection

Peripheral blood samples were collected from all MS-affected individuals and healthy subjects using EDTA anticoagulant vacuum blood collection tubes early in the morning. The mixture was gently mixed by inverting it six to ten times to ensure that the blood would not clot. Subsequently, blood samples were centrifuged at 3500 g for 10 min at 4°C, and the plasma was transferred to new centrifuge tubes and quickly frozen in liquid nitrogen. Immediately, all separated plasma was stored at -80°C for future study.

### 2.3 Global Untargeted Metabolomics Analysis

#### 2.3.1 Preparation of NIST Serum Standard Curve Correction Solutions

Initially, 5, 10, 50, 100, 200, and 300 μL NIST serum (National Institute of Standards and Technology, Standard Reference Material^®^ 1950) was added to centrifuge tubes, respectively, followed by the supplement of ddH2O, mixed internal standard solution, and methanol solution (ThermoFisher Scientific, USA) to a certain proportion. The six NIST serum samples were thoroughly vortexed. Samples were centrifuged at 13 500 ×g and 4°C for 10 min; then, the supernatant from each sample was transferred to another 2 mL centrifuge tube. Subsequently, samples were concentrated to dryness in vacuum before dissolving in 150 μL of 80% methanol (precooled at -20°C), then centrifuged again at 13 500 ×g and 4°C for 10 min to obtain the supernatant for high-performance liquid chromatography Ultimate 3000 (Thermo Fisher Scientific, USA)-tandem mass spectrometry Q Exactive Focus (Thermo Fisher Scientific, USA) (LC-MS/MS) analysis (95, 96).

#### 2.3.2 Extraction of Total Metabolites

All samples were thawed at 4°C, and 100 μL of each sample were pipetted to new 2 mL tubes. Then, 100 μL mixed internal standard solution and 400 μL methanol solution (precooled at -20°C) were added to each sample and vortexed thoroughly for 60 s. Samples were then centrifuged at 13 500 ×g and 4°C for 10 min. Subsequently, 500 μL supernatant from each sample was pipetted to another 2 mL tube. Samples were concentrated to dryness in vacuum before dissolving in 150 μL 80% methanol solution, then centrifuged again at 13 500 ×g and 4°C for 10 min, and the supernatant was transferred to a new tube for LC-MS detection. Quality control (QC) samples were used for the monitoring of any deviations in the analytical results (97, 98).

### 2.4 Chromatographic Assay

A chromatographic separation assay was performed using the Thermo Ultimate 3000 platform coupled with an ACQUITY UPLC^®^ HSS T3 column (2.1 × 150 mm × 1.8 μm; Waters Co. Ltd., Milford, MA, USA). The temperature of the automatic sampler was set to 8°C. Gradient elution of analytes was carried out with 0.1% formic acid (TCI Co. Ltd., Tokyo, Japan), inddH2O (Millipore), and 0.1% formic acid in acetonitrile (ThermoFisher Scientific, USA) or 5.0 mM ammonium formate (Sigma) in ddH2O and acetonitrile at a flow rate of 0.25 mL/min. Injection of 2.0 μL of each sample was performed after equilibration.

### 2.5 Mass Spectrometry Detection

Detection of metabolites was performed using a Thermo Q Exactive Focusmass spectrometer (ThermoFisher Scientific, USA) with both positive and negative ESI models, respectively. Simultaneous MS1 and MS/MS (full MS mode with data-dependent acquisition, DDA) was employed. The parameters were as follows: sheath gases pressure, 30 arbitrary (arb) units, auxiliary gases pressure, 10 arb; spray voltage, 3.50 kV for ESI (+), and -2.50 kV for ESI (-); capillary temperature, 325°C; MS1 scan range, mass/charge (*m/z*) 81-1000; MS1 resolving power, 70 000 FWHM; number of scans per cycle, 3; MS/MS resolving power, 17 500 FWHM; normalized collision energy, 30 eV; dynamic exclusion time, automatic.

### 2.6 Data Acquisition

The components were separated using chromatography, followed by mass spectrometry analysis for data acquisition. Each scan produced a mass spectrogram, and the ion with the highest intensity was continuously described with the ion strength as the ordinate and time as the abscissa; thus, the obtained map was the base peak chromatogram (BPC).

### 2.7 Data Processing and Data Quality Control

#### 2.7.1 Data Preprocessing

The raw data were transformed into the mzXML format by the Proteowizard software (v3.0.8789). The identification, filtration, and alignment of peaks were accomplished using the XCMS package of R (v.3.3.2). Then, the data matrix, including the *m/z* ratio, retention time, and relative ratio of the peak area were acquired. A total of 13462 and 16359 precursor molecules were acquired from the positive and negative ion models, respectively, which were used for subsequent analysis.

#### 2.7.2 Relative Quantitative Analysis

Whole target relative quantitative analysis is a canonical method based on UPLC/HRMS metabolomics, which obtains the original mass spectrometry data through the preparation of mixed solutions of an internal isotope standard, correction solutions for the standard curve, and sample solutions. The relative quantification of certain metabolites in sample solutions was done by fitting a linear equation and optimizing the isotope internal standard.

### 2.8 Identification of Isotope Internal Standards

Cholic acid-D5, phenylalanine-D5, methionine-D4, tryptophan-D3, and choline-D9 were identified as isotopic internal standards in the positive ion mode, whereas succinic acid-D4, cholic acid-D5, phenylalanine-D5, methionine-D4, and tryptophan-D3 were identified in the negative ion mode.

#### 2.8.1 Linear Equation Fitting and Isotope Internal Standard Optimization

For each first-order variable in the sample, the peak area of the corresponding first-order variable in the standard curve correction sample was calculated. In addition, the ratio of the isotope internal standard peak area identified in the positive and negative ion models to the corrected concentration of the standard curve was linearly fitted. Finally, the isotope internal standard with the largest correlation coefficient (R) of the linear equation was selected as the preferred linear equation.

#### 2.8.2 Calculation of the Quantitative Concentration of First-Order Variables

The peak area ratio of a metabolite to that of the isotope internal standard was substituted into the optimal linear equation obtained in the previous step. The relative quantitative concentrations of metabolites in the sample were calculated. After screening the results of the relative quantitative analysis, a total of 5683 and 4491 precursor molecules were acquired from the positive and negative ion models, respectively. To compare the data of different orders of magnitude, the peak areas of the data were subjected to batch normalization.

To identify biomarkers, the relative standard deviation (RSD) of a characteristic peak in the QC sample must not exceed 30%. Otherwise, the corresponding peaks should be removed. Therefore, on the basis of QC, quality assurance is usually performed to delete characteristic peaks with poor repeatability in QC samples, thus obtaining higher quality datasets, which are more conducive to the detection of biomarkers. The percentage of peaks characterized by an RSD value < 30% in our QC samples reached approximately 70%, indicating that the data were good.

### 2.9 Bioinformatic Analysis

#### 2.9.1 Agglomerate Hierarchical Clustering

We employed an agglomerate hierarchical clustering method to classify each object into a certain class and merge these classes into increasingly larger classes. The relative quantitative levels of metabolites were determined using the Pheatmap package of R (v.3.3.2). All samples and related data were calculated using a distance matrix and clustered using the average-linkage clustering method.

#### 2.9.2 Multivariate Statistical Analysis

Metabolomic data are typically converted into appropriate weights before multivariate statistical analysis, a process called scaling processing. In this study, we first performed an autoscaling method before performing multivariate statistical analysis to acquire more intuitive and reliable results. The multivariate statistical analysis (R language Ropls package) methods adopted in this experiment were as follows: unsupervised analysis, such as principal component analysis (PCA), and supervised analysis, such as orthogonal partial least squares discriminant analysis (OPLS-DA) and partial least squares-discriminant analysis (PLS-DA).

#### 2.9.3 Identification of Differentially Abundant Metabolites

Differentially abundant metabolites were identified using parameters with variable importance for the projection (VIP) ≥ 1.00 and a *P* value < 0.05. During the process of the identification of metabolites, we first confirmed the exact molecular weight (MW) of metabolites (MW error < 15 ppm), and then acquired the accurate information of metabolites by further matching and annotating them in the Human Metabolome Database (HMDB) (http://www.hmdb.ca), Metlin (https://metlin.scripps.edu), massbank (http://www.mzcloud.org), LipidMaps (http://www.lipidmaps.org), mzclound (https://www.mzclound.org), and MoNA (https://mona.fiehnlab.ucdavis.edu). Moreover, the standard database was built by BioNovoGene Co., Ltd. (Suzhou, China), according to the fragment information obtained by the MS/MS mode. Subsequently, all identified metabolites were classified using the Kyoto Encyclopedia of Genes and Genomes (KEGG) and Metabolon databases. In the analysis of differential metabolites, the standard score (Z-score) was transformed based on the relative content of metabolites (99), which was used to measure the relative content of metabolites at the same level. The Z-score was calculated based on the average and standard deviation of the reference dataset (control group), according to z = (x-μ)/σ, where x indicates a specific score, μ denotes the average, and σ represents the standard deviation. A heatmap was plotted using agglomerate hierarchical clustering. The correlation between metabolites was analyzed by calculating the Pearson correlation coefficient using the cor () function in R (v.3.1.3). Meanwhile, the cor. test () function of R (v.3.1.3) was used to analyze the correlation between metabolites, with a significant correlation set when *P* < 0.05 (100). The MetPA database, a part of MetaboAnalyst (www.metaboanalyst.ca), was employed to analyze the related metabolic pathways of the two groups of differential metabolites. The algorithm used for data analysis was a hypergeometric test, and the topology of the pathway was determined by the relative betweenness centrality. Based on the above MetPA analysis results, and according to the relative response values of metabolites identified in metabolic pathways and the dimensionality reduction algorithm, the relative response values of metabolic pathways were obtained. The correlation coefficients between metabolic pathways were subsequently calculated, allowing us to draw an association network diagram of metabolic pathways.

### 2.10 Measurement of Levels of Immunoregulatory Factors

The magnetic beads immunoassay kit (Bio-Rad, CA, USA) combined with the Bio-Plex 200 system was applied to quantify the concentrations of the following cytokines and chemokines: Tumor necrosis factor-α (TNF-α), Interleukin-17 (IL-17), IL-7, IL-8, IL-9, IL-12, IL-13, IL-1 receptor antagonist protein (IL-1ra), Eotaxin, Interferon gamma-inducible protein 10 (IP-10), Interferon gamma (IFN-γ), Monocyte chemotactic protein-1 (MCP-1), Platelet-derived growth factor (PDGF-bb), Macrophage inflammatory protein-1α (MIP-1α), MIP-1β, and Regulated upon activation normal T-cell expressed and secreted (RANTES). Detailed experimental procedures and quantitation methods were described in a previous study (101).

### 2.11 Statistical Analyses

Independent *t*-tests, White’s nonparametric *t*-tests, and Mann-Whitney *U*-tests were used to analyze continuous variables. Benjamini-Hochberg (BH) procedure was used for correction and obtained adjusted *P* (*Padj*) value. The Pearson chi-square test was applied to analyze categorical variables between groups, depending on the validity of assumptions. The Pearson correlation coefficient was calculated to determine the correlation between the levels of differential metabolites and immunoregulatory factors. SPSS V19.0 (Chicago, IL, USA) was used to perform statistical analysis. GraphPad Prism (San Diego, CA, USA) was used to prepare graphs. Two-sided statistical significance was tested, and only a *P value* < 0.05 or a corrected *P value* < 0.05 was interpreted as statistically significant.

## 3 Results

### 3.1 Identification of Differentially Abundant Metabolites in Patients With Multiple Sclerosis

**Figure 1:**
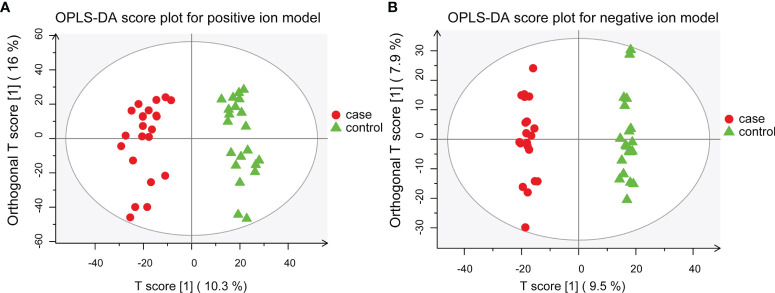
OPLS-DA models for separating patients with MS and healthy controls. **(A)** OPLS-DA plot for positive ion model. The respective model interpretability for the X and Y variable datasets was R2X = 0.263 and R2Y = 0.945, model predictability Q2 = 0.868. **(B)** OPLS-DA plot for the negative ion model. The respective model interpretability for the X and Y variable datasets was R2X = 0.216 and R2Y = 0.993, model predictability Q2 = 0.797.

To further dissect the characteristics of the metabolic landscape in Chinese patients with MS, we performed a global untargeted metabolomics analysis using the plasma samples of 22 patients with MS, including 20 RRMS and 2 PPMS cases, and those from 21 sex- and age-matched healthy controls (**Table 1**). Our results from both the supervised, including OPLS-DA and PLS-DA, and unsupervised analyses (i.e., PCA) revealed a proper separation of all patients with MS from healthy controls in either positive or negative ionization mode (**Figures 1A, B**, **S1A–F**), suggesting a significant alteration of the metabolic profile in the peripheral system of Chinese patients with MS. In addition, we found that the most populated category among all classified identified metabolites was “amino acid” (33.0%), followed by “lipid” (28.0%), “cofactors and vitamins” (11.0%), “nucleotide” (10.0%), “carbohydrate” (9.0%), and “xenobiotics” (8.0%) (**Figure S2**).

**Figure 2:**
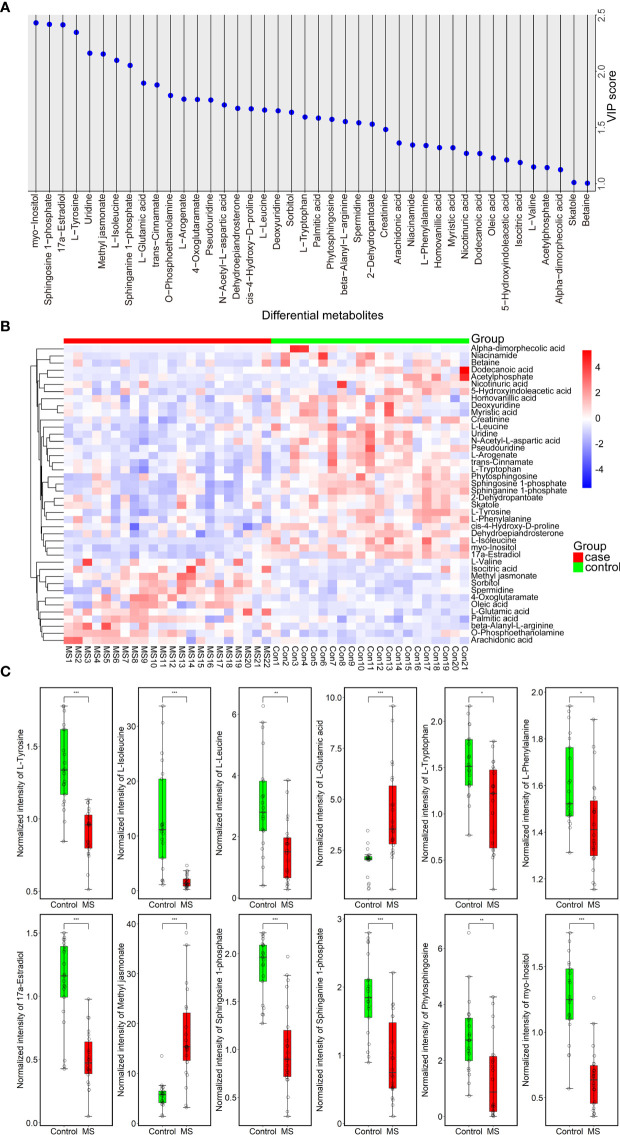
Forty-two metabolites with significant differences between MS-affected patients and healthy subjects. **(A)** VIP plot of a total of 42 DAMs. VIP > 1.000, *P* value < 0.05. **(B)** Heatmap of all DAMs; the relative quantitative level of metabolites was determined using the Pheatmap package in R (v.3.3.2); red and blue denote up- and downregulated, respectively. Case group: MS 1-22, control group: Con 1-21. **(C)** Normalized intensity of 12 representative DAMs belonging to amino acids, lipids, and carbohydrates in the plasma samples of 22 patients with MS and 21 healthy controls. Samples were compared using the T-test. *P* value was corrected using Benjamini-Hochberg procedure. *Padj < 0.05, **Padj < 0.01, ***Padj < 0.001.

Using LC-MS/MS analysis, we identified a total of 42 differentially abundant metabolites (DAMs) in patients with MS (**Figure 2A** and **Tables S1**, **S2**). Further analysis revealed a significant increase and decrease in the relative levels of 12 and 30 differential metabolites, respectively, in patients with MS as compared with healthy subjects (**Figures 2B** and **S3**). We observed that the relative levels of several amino acid metabolites, such as L-tyrosine (VIP = 2.345; *Padj* = 5.95e-05), L-tryptophan (VIP = 1.593; *Padj* = 1.50e-02), L-phenylalanine (VIP = 1.340; *Padj* = 3.30e-02), L-leucine (VIP = 1.654; *Padj* = 4.90e-03), and L-isoleucine (VIP = 2.096; *Padj* = 9.50e-05), were prominently reduced in MS-affected subjects compared with those in healthy controls (**Figure 2C**). In contrast, the relative levels of L-glutamic acid (VIP = 1.894; *Padj* = 3.27e-04) were markedly increased in patients with MS (**Figure 2C**), consistent with the findings of a previous study (26). Moreover, we found that the relative levels of lipid metabolites, such as sphingosine 1-phosphate (VIP = 2.417; *Padj* = 7.41e-05), sphinganine 1-phosphate (VIP = 2.051; *Padj* = 5.10e-04), phytosphingosine (VIP = 1.572; *Padj* = 8.71e-03), and 17a-estradiol (VIP = 2.412; *Padj* = 2.65e-04) were drastically decreased in patients with MS (**Figure 2C**) compared with those in healthy controls, whereas the relative level of methyl jasmonate (VIP = 2.152; *Padj* = 1.63e-04), a linolenic acid metabolite, was significantly increased in patients with MS (**Figure 2C**). In addition, the level of the carbohydrate metabolite myo-inositol (VIP = 2.430; *Padj* = 6.15e-05) was also greatly decreased in MS-affected individuals compared with that in healthy subjects (**Figure 2C**).

We also observed a significant elevation in the circulating levels of fatty acids, such as oleic acid (VIP = 1.229; *Padj* = 4.58e-02), palmitic acid (VIP = 1.583; *Padj* = 8.05e-03), and arachidonic acid (VIP = 1.362; *Padj* = 1.50e-02), as well as in those of several amino acid metabolites, such as beta-alanyl-L-arginine (VIP = 1.552; *Padj* = 5.61e-03) and L-valine (VIP = 1.149; *P* = 1.56e-02), in the plasma of patients with MS compared with that in healthy subjects (**Figure S3B**). In addition, we noticed that the levels of 4-oxoglutaramate (VIP = 1.748; *Padj* = 4.90e-03), isocitric acid (VIP = 1.189; *Padj* = 1.87e-02), O-phosphoethanolamine (VIP = 1.784; *Padj* = 2.84e-03), sorbitol (VIP = 1.635; *Padj* = 9.30e-03), and spermidine (VIP = 1.542; *Padj* = 1.25e-02) were also significantly increased in MS-affected patients relative to those in healthy subjects (**Figure S3B**). We also found an extensive reduction in the concentrations of a large proportion of DAMs, such as dehydroepiandrosterone (VIP = 1.670; *Padj* = 1.14e-02), 2-dehydropantoate (VIP = 1.529; *Padj* = 1.11e-02), skatole (3-methylindole) (VIP = 1.011; *Padj* = 9.95e-02), 5-hydroxyindoleacetic acid (VIP = 1.211; *Padj* = 9.07e-02), cis-4-hydroxy-D-proline (VIP = 1.666; *Padj* = 4.10e-03), myristic acid (VIP = 1.319; *Padj* = 4.81e-02), N-acetyl-L-aspartic acid (VIP = 1.700; *Padj* = 7.24e-03), homovanillic acid (VIP = 1.320; *Padj* = 2.34e-02), creatinine (VIP = 1.482; *Padj* = 1.05e-02), nicotinuric acid (VIP = 1.269; *Padj* = 2.49e-02), trans-cinnamate (VIP = 1.878; *Padj* = 3.32e-03), deoxyuridine (VIP = 1.648; *Padj* = 5.97e-03), L-arogenate (VIP = 1.751; *Padj* = 1.27e-02), betaine (VIP = 1.005; *Padj* = 9.07e-02), pseudouridine (VIP = 1.744; *Padj* = 3.79e-03), acetylphosphate (VIP = 1.144; *Padj* = 6.16e-02), uridine (VIP = 2.160; *Padj* = 2.86e-04), dodecanoic acid (VIP = 1.268; *Padj* = 1.51e-02), niacinamide (VIP = 1.344; *Padj* = 3.87e-02), and alpha-dimorphecolic acid (VIP = 1.124; *Padj* = 7.64e-03), in MS-affected patients compared with those in healthy controls (**Figure S3B**). However, the adjusted *P* value of L-valine, skatole, 5-hydroxyindoleacetic acid, betaine, and acetylphosphate was not significant (*Padj* > 0.05) (**Table S2**).

### 3.2 Correlation and Functional Pathway Analysis of Differentially Abundant Metabolites

**Figure 3:**
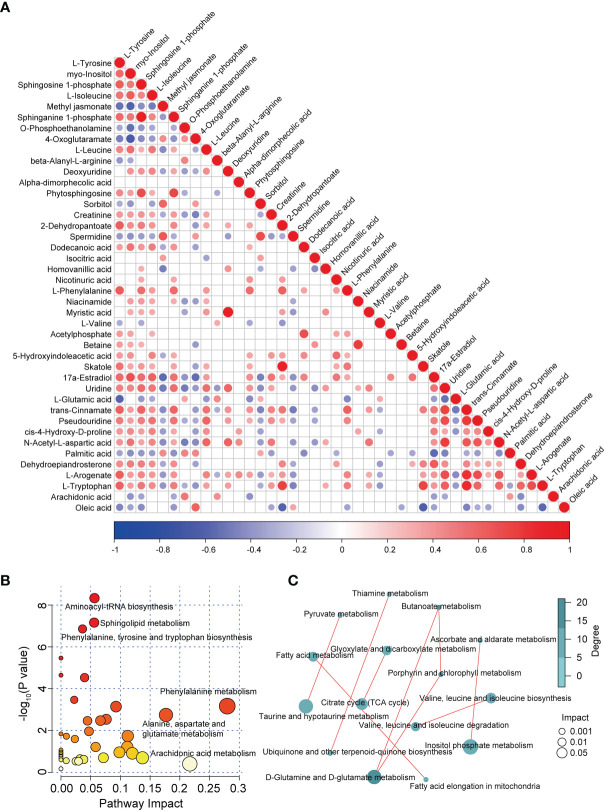
Correlation and pathway analysis of DAMs. **(A)** Correlation heatmap of all DAMs. Correlation analysis was conducted by calculating the Pearson’s correlation coefficient of two metabolites; positive and negative correlation is represented by red and blue, respectively. **(B)** Bubble diagram of enriched pathways of all DAMs in patients with MS. **(C)** Correlation analysis of enriched pathways in patients with MS.

We performed correlation analysis on the alterations in the levels of differentially abundant metabolites. Notably, we observed a significant positive correlation between changes in the levels of sphinganine 1-phosphate and sphingosine 1-phosphate (correlation coefficient = 0.9270; *P* < 1.00e-15), myristic acid and deoxyuridine (correlation coefficient = 0.9518; *P* < 1.00e-15), skatole and 2-dehydropantoate (correlation coefficient = 0.8908; *P* = 1.33e-15), L-tryptophan, and trans-cinnamate (correlation coefficient = 0.8908; *P* = 9.48e-13), as well as L-arogenate and trans-cinnamate (correlation coefficient = 0.8145; *P* = 3.05e-11; **Figure 3A**). In contrast, we identified an evident negative correlation between changes in the levels of 4-oxoglutaramate and myo-inositol (correlation coefficient = -0.6528; *P* = 2.09e-06), oleic acid and skatole (correlation coefficient = -0.6141; *P* = 1.19e-05), methyl jasmonate and myo-inositol (correlation coefficient = -0.5899; *P* = 3.15e-05), as well as oleic acid and 2-dehydropantoate (correlation coefficient = -0.5875; *P* = 3.44e-05; **Figure 3A**). In addition, an internal interaction and crosstalk network was also established between amino acids, lipids, cofactors and vitamins, nucleotides, and carbohydrate metabolites (**Figure S4A**). Linkages of representative DAMs, such as sphingolipid metabolites and the metabolism products of tryptophan, are shown in **Figure S4B**.

To investigate the disturbed metabolic pathways in patients with MS, we performed pathway enrichment analysis on all DAMs using the KEGG database. As shown in the bubble plot in **Figure 3B**, the most enriched pathways in patients with MS were those of amino acid and lipid metabolism, such as “aminoacyl-tRNA biosynthesis”, “phenylalanine, tyrosine, and tryptophan biosynthesis”, “phenylalanine metabolism”, “alanine, aspartate, and glutamate metabolism”, “sphingolipid metabolism”, and “arachidonic acid metabolism” (**Figures 3B** and **S4C**). Moreover, we detected various correlations between these enriched pathways. For instance, we observed tight correlations between the metabolism of D-glutamine and D-glutamate and the metabolism of butanoate, taurine, and hypotaurine, and pyruvate, inositol phosphate metabolism and ascorbate and aldarate metabolism, as well as between fatty acid elongation in mitochondria and fatty acid metabolism, valine, leucine, and isoleucine biosynthesis and their degradation, and the citrate cycle (TCA cycle) and glyoxylate and dicarboxylate metabolism (**Figure 3C**).

### 3.3 Altered Immune and Inflammation Responses In Patients With Multiple Sclerosis

**Figure 4:**
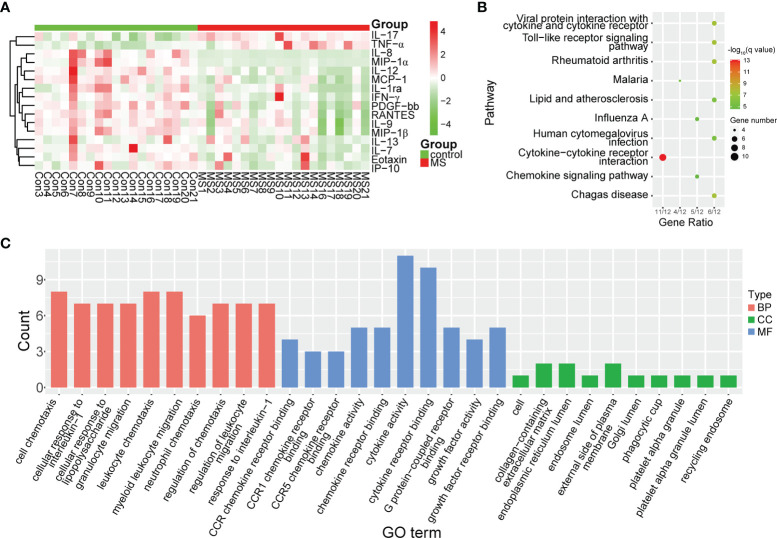
Differential expression of cytokines and chemokines in MS-affected patients. **(A)** Heatmap plot of differentially expressed cytokines and chemokines. The levels of proinflammatory cytokines, such as IL-17 and TNF-α, were significantly increased, whereas those of chemokines, such as IL-8, MIP-1α and MIP-1β, were drastically decreased in patients with MS. **(B, C)** Enrichment analysis of pathway and gene ontology (GO) of differentially expressed cytokines and chemokines. BP, biological process; CC, cellular compartment; MF, molecular function.

**Figure 5:**
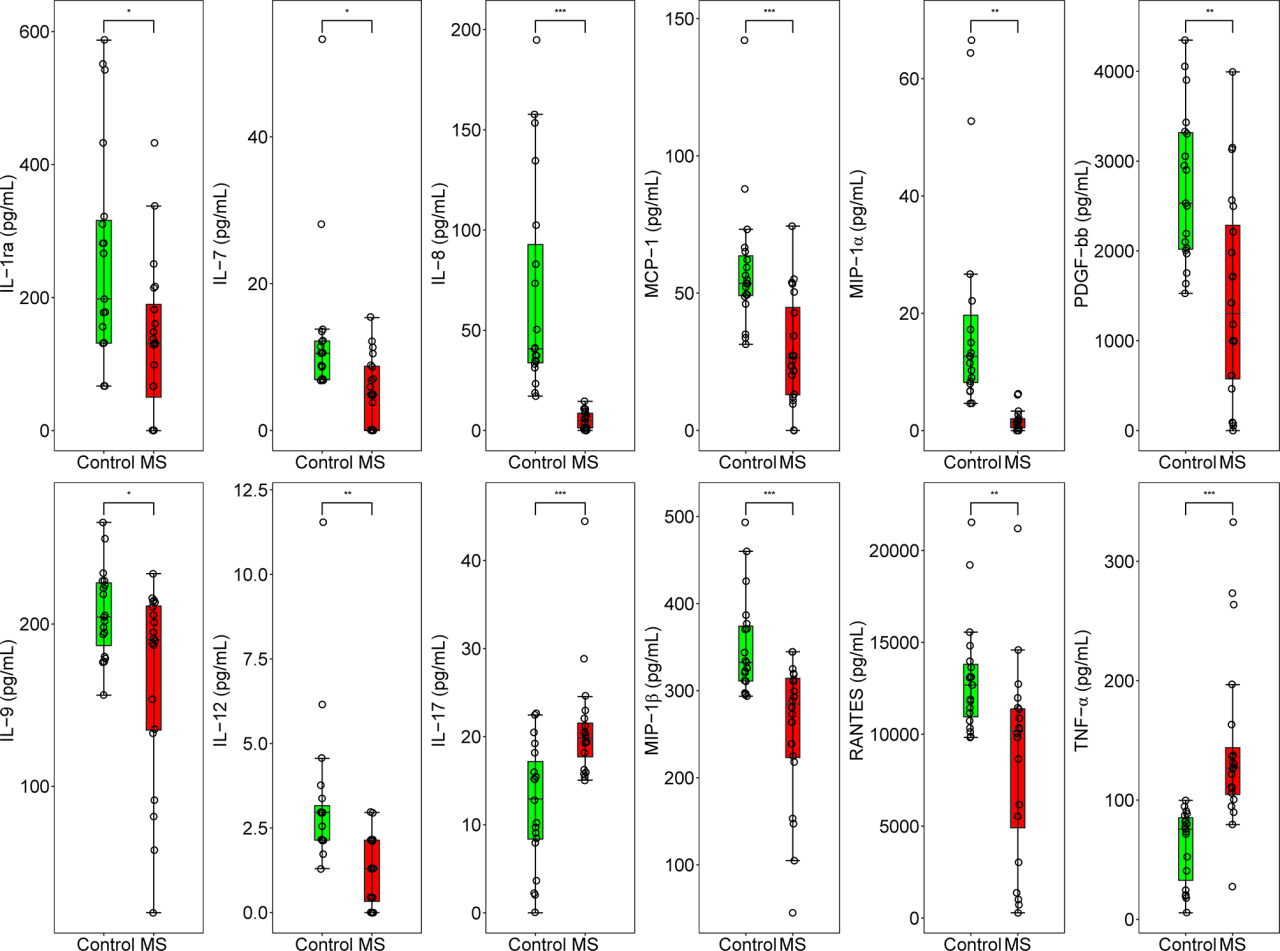
Concentration of 12 inflammation regulatory cytokines and chemokines in MS-affected patients and healthy subjects. Quantitative results of the concentration of IL-1ra, IL-7, IL-8, MCP-1, MIP-1α, PDGF-bb, IL-9, IL-12, IL-17, MIP-1β, RANTES, and TNF-α (pg/mL) in the plasma samples of MS-affected patients and healthy controls. Samples were compared using the T-test. *P* value was corrected using Benjamini-Hochberg procedure. *Padj < 0.05, **Padj < 0.01, ***Padj < 0.001.

To determine whether the levels of cytokines and chemokines changed in MS-affected patients, we measured the levels of 16 representative cytokines and chemokines, such as IL-17 and TNF-α. We found that the levels of all analyzed cytokines and chemokines were extensively altered in patients with MS compared with those in healthy subjects (**Figure 4A**). We observed that the most enriched pathways were those of immune responses, such as cytokine-cytokine receptor interactions and the chemokine signaling pathway (**Figure 4B**). Our results from gene ontology (GO) analysis indicated that the majority of these differentially expressed cytokines and chemokines participated in immuno-inflammatory processes, such as cellular responses to interleukin-1 and lipopolysaccharides, leukocyte and neutrophil chemotaxis, and regulation of leukocyte migration (**Figure 4C**). Moreover, we found that the main functions of these immuno-inflammatory factors were binding to the CCR chemokine receptor and regulating cytokine activity (**Figure 4C**). We noticed that compared with healthy controls, the contents of proinflammatory cytokines, such as IL-17 and TNF-α, were significantly increased (*Padj* < 0.001), whereas those of IL-12 were prominently decreased (*Padj* < 0.01) in patients with MS (**Figure 5**). Furthermore, we detected that the concentration of anti-inflammatory mediators, such as IL-1ra, and several chemokines, such as IL-7, IL-8, RANTES, MIP-1α, MIP-1β, and MCP-1, was significantly decreased in patients with MS (*Padj* < 0.05) (**Figure 5**). The levels of pleiotropic cytokine IL-9 and growth factor PDGF-bb were also markedly decreased in patients with MS (*Padj* < 0.05) (**Figure 5**). These results were consistent with previously obtained data (101). Nevertheless, we noticed that the levels of IL-13, IP-10, IFN-γ, and Eotaxin showed no significant change in patients with MS (*P* > 0.05) (**Table S3**), which might be attributed to individual heterogeneity.

### 3.4 Correlations Between Differentially Abundant Metabolites and Inflammation Among Multiple Sclerosis-Affected Patients

**Figure 6:**
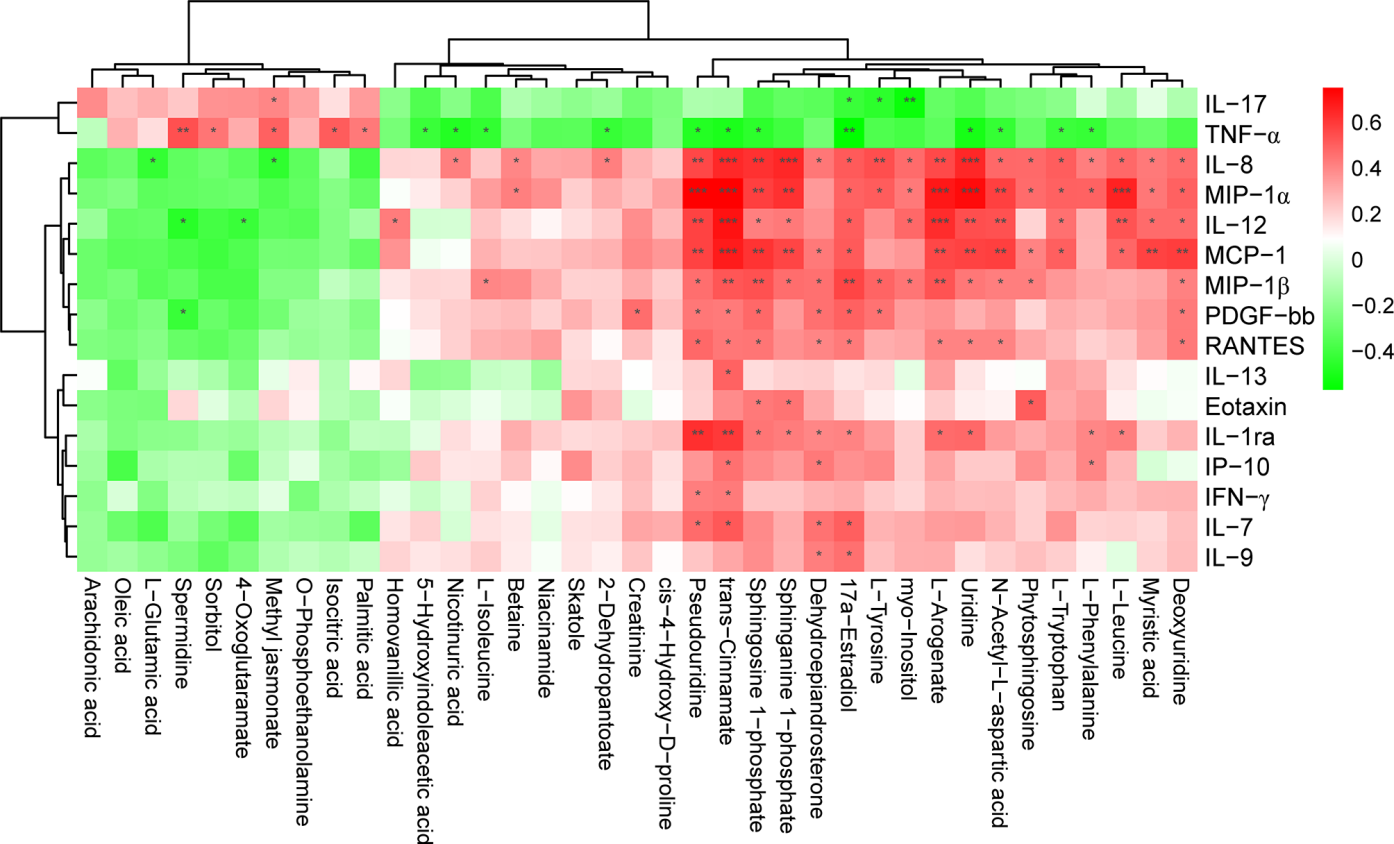
Significant correlation between the level of cytokines, chemokines, and DAMs in patients with MS. The heatmap was plotted using Pearson’s correlation analysis. The correlation coefficient is denoted by red and blue, representing positive and negative correlations, respectively. Asterisks indicate significant positive or negative correlations. *P* value was corrected using Benjamini-Hochberg procedure. *Padj < 0.05; **Padj < 0.01; ***Padj < 0.001.

We further evaluated the correlations between changes in the levels of cytokines and chemokines and the levels of DAMs among patients with MS. We found that upregulated metabolites, such as palmitic acid, isocitric acid, methyl jasmonate, sorbitol, and spermidine, positively correlated with proinflammatory cytokines, such as IL-17 and TNF-α, while they negatively correlated with various chemokines, such as IL-8, IL-12, and PDGF-bb (**Figure 6**). In contrast, we observed that the decreased levels of metabolites such as L-tryptophan and sphingosine 1-phosphate were negatively correlated with changes in the levels of TNF-α and IL-17, but positively correlated with reduced levels of chemokines, such as MIP-1α and IL-8 (**Figure 6**). We also found that some differential metabolites like trans-cinnamate and sphingosine 1-phosphate was significantly correlated with IL-13, IP-10, IFN-γ, and Eotaxin (*Padj* < 0.05), respectively, despite the lack of any obvious change in the levels of these chemokines in patients with MS (**Figure 6**). Moreover, we noticed that even certain metabolites with no significant changes in their levels (*P* > 0.05), such as 3-hydroxyphenylacetate and prostaglandin H2, were also significantly correlated with IL-12, MCP-1 and IL-7 among patients with MS (*Padj* < 0.05) (**Figure S5**), respectively. However, several differential metabolites with observable changes in their levels (*P* < 0.05), such as L-valine, beta-alanyl-L-arginine, alpha-dimorphecolic acid, dodecanoic acid, and acetylphosphate, showed no significant correlation with the above pro- and anti-inflammatory factors (*P* > 0.05) (**Figure S6**). These results indicated that the disturbed profile of plasma metabolites was strongly associated with the altered concentrations of cytokines and chemokines, implying that altered metabolic profiles might affect the pathogenesis of MS by modulating the immuno-inflammatory responses in the peripheral system.

### 3.5 Sphingolipid Metabolites, L-Tryptophan, and Myo-Inositol Significantly Correlated With Inflammation Responses

**Figure 7:**
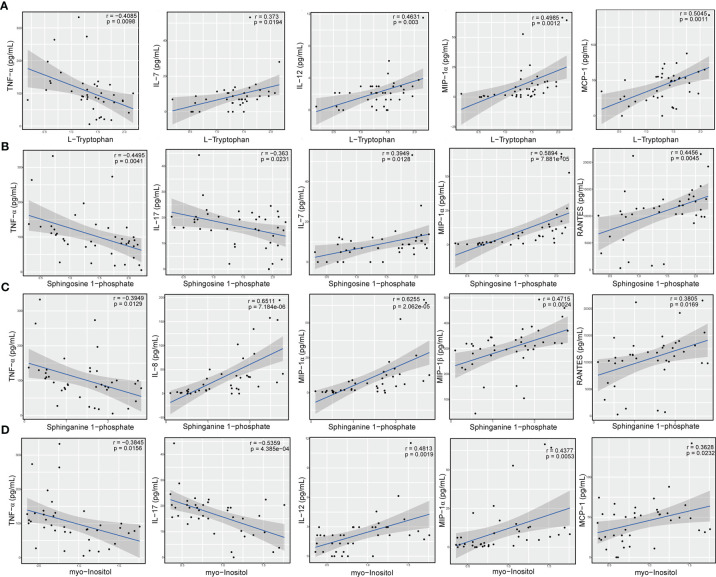
Levels of amino acid, sphingolipid, and carbohydrate metabolites significantly correlated with concentration of cytokines and chemokines. Correlation between the relative level of L-tryptophan and the concentrations of TNF-α, IL-7, IL-12, MIP-1α, and MCP-1 **(A)**, relative levels of sphingosine 1-phosphate and the concentrations of TNF-α, IL-17, IL-7, MIP-1α, and RANTES **(B)**, relative levels of sphinganine 1-phosphate and contents of TNF-α, IL-8, MIP-1α, MIP-1β, and RANTES **(C)**, and relative levels of myo-inositol and levels of TNF-α, IL-17, IL-12, MIP-1α, and MCP-1 **(D)**. The statistical importance was evaluated by Pearson’s correlation (r) and probability (p). The gray area around the blue straight line denotes the 95% confidence interval.

To further determine whether certain differential metabolites were significantly associated with pro- and anti-inflammatory cytokines and chemokines, we performed Pearson correlation analysis on all DAMs and on the changes in the levels of cytokines and chemokines. In particular, we observed that L-tryptophan showed a negative correlation with the level of TNF-α (r = -0.4085; *P* = 9.80e-03) (**Figure 7A**). In contrast, we found that the concentrations of IL-7 (r = 0.3730; *P* = 1.94e-02), IL-12 (r = 0.4631; *P* = 3.00e-03), MIP-1α (r = 0.4985; *P* = 1.20e-03), and MCP-1 (r = 0.5045; *P* = 1.10e-03) were positively associated with those of L-tryptophan (**Figure 7A**). Moreover, the levels of the sphingolipid metabolite, sphingosine 1-phosphate, showed an obvious negative correlation with the concentrations of TNF-α (r = -0.4495; *P* = 4.10e-03) and IL-17 (r = -0.3630; *P* = 2.31e-02) (**Figure 7B**), whereas it positively correlated with the concentrations of several chemokines, such as IL-7 (r = 0.3949; *P* = 1.28e-02), MIP-1α (r = 0.5894; *P* = 7.88e-05), and RANTES (r = 0.4456; *P* = 4.50e-03) (**Figure 7B**). Similarly, we found that the level of sphinganine 1-phosphate was negatively correlated with the contents of proinflammatory cytokines, such as TNF-α (r = -0.3949; *P* = 1.29e-02), whereas it was positively correlated with the level of chemokines, such as IL-8 (r = 0.6511; *P* = 7.18e-06), MIP-1α (r = 0.6255; *P* = 2.06e-05), MIP-1β (r = 0.4715; *P* = 2.40e-03), and RANTES (r = 0.3805; *P* = 1.69e-02) (**Figure 7C**). In addition, we also observed that the level of myo-inositol was negatively correlated with the levels of proinflammatory cytokines, such as TNF-α (r = -0.3845; *P* = 1.56e-02) and IL-17 (r = -0.5359; *P* = 4.39e-04) (**Figure 7D**), while it was positively correlated with the concentration of IL-12 (r = 0.4813; *P* = 1.90e-03), MIP-1α (r = 0.4377; *P* = 5.30e-03), and MCP-1 (r = 0.3628; *P* = 2.32e-02) (**Figure 7D**). These results suggested that alterations in amino acids, sphingolipids, and carbohydrates, such as L-tryptophan, sphingosine 1-phosphate, and myo-inositol, were tightly correlated with immunoinflammatory changes in the circulatory system of Chinese patients with MS.

## 4 Discussion

Mounting evidence indicates the important role of the complex interaction between metabolic networks and the immune system in modulating autoimmunity responses (50–53) and in facilitating the etiopathology of multiple conditions such as cancer and neurodegenerative diseases (54, 55). In particular, multiple studies have demonstrated that amino acid metabolites, such as tryptophan, play an indispensable role in regulating immune homeostasis in MS (79–83) and in an EAE model (91–93). A recent study demonstrated that SCFAs, such as propionic acid, were capable of restoring the Treg cell/Th17 imbalance and improving the course of MS (65). Numerous studies have suggested that sphingolipid metabolites function as key regulators in the T-cell lineage specification and immune responses in inflammatory diseases (58–61, 63), including MS and EAE (56, 57, 62, 64).

To date, the majority of metabolomic studies on MS have primarily focused on Caucasian populations (20–41, 44), which largely differ from Chinese patients with MS. Studies on specific populations could lead to a failed validation of potential metabolite biomarkers in MS, thus impeding their diagnostic and therapeutic clinical application. Multiple factors, such as ethnic background, environmental conditions, dietary habits, and lifestyle, can influence the metabolic profile in the peripheral blood, cerebrospinal fluid (CSF), and urine. In particular, the dietary pattern of the Chinese population, which is characterized by a reduced consumption of fat and calories but an increased intake of dietary fibers, is expected to alter the metabolomic profile of the population, potentially interfering with the progression of MS. Given that different populations show diverse metabolic landscapes, we speculated that specific alterations might characterize the metabolic profiles of the peripheral system in Chinese patients with MS. Consequently, these altered metabolic profiles might modulate the circulating immuno-inflammatory responses that accelerate inflammation in the CNS.

In the current study, we found that the metabolic profile in the peripheral blood of Chinese patients with MS significantly differed from that of healthy controls. Among all DAMs, the most significant alterations were observed in the levels of amino acid metabolites; for instance, the relative levels of beta-alanyl-L-arginine, L-glutamic acid, and L-valine were significantly increased, whereas those of L-tryptophan, L-leucine, L-isoleucine, L-tyrosine, L-phenylalanine, and cis-4-hydroxy-D-proline were significantly reduced in MS-affected patients compared with those in healthy subjects. Independent studies also showed similar changes in amino acid profiles in MS-affected subjects (102, 103). The upregulation in the levels of L-glutamic acid and the downregulation in the levels of L-leucine and L-isoleucine were consistent with the results of previous studies (26, 44). In addition, the reduced levels of tyrosine and proline were also in line with the metabolomic results obtained from an EAE model (104). However, some studies reported that the level of phenylalanine was significantly increased in the CSF of MS-affected patients (105), whereas the concentration of arginine in the serum and CSF showed no obvious differences between patients with MS and healthy subjects (38), which contradicted our results. The inconsistent outcomes of different studies could be attributed to the type of detected biospecimens (for example, CSF versus plasma), and the types of investigated populations (i.e., ethnic background heterogeneity). L-tryptophan functions as a crucial mediator in modulating immune homeostasis in MS (80–83). Previous study suggested that the decreased level of serum tryptophan was associated with the risk and course of pediatric MS disease (106). Interestingly, we also observed a distinct reduction in the level of L-tryptophan in this study. This implies that a reduced level of L-tryptophan might aggravate peripheral immunoinflammatory responses *via* the AHR-mediated signaling in MS-affected patients.

Additionally, the levels of lipid metabolites also significantly differed between patients with MS and healthy subjects. For instance, the relative levels of sphingosine 1-phosphate, phytosphingosine, sphinganine 1-phosphate, and 17a-estradiol were greatly decreased, whereas those of methyl jasmonate were distinctly increased in MS-affected patients compared with healthy subjects. Sphingolipid metabolites, such as sphingosine 1-phosphate, play a crucial role in the modulation of inflammatory diseases (57–64) as well as in regulating inflammation and neurodegeneration in EAE (56). Recent study showed significant changes in sphingolipid metabolism in patients with MS (107). In this study, we also observed an extensive alteration in the levels of sphingolipid metabolites in patients with MS, including sphingosine 1-phosphate, phytosphingosine, and sphinganine 1-phosphate, suggesting that the process of inflammatory responses might be changed by altered sphingolipid metabolites in Chinese patients with MS. Moreover, it is well known that estrogen reinforces pleiotropic neuroprotective effects on the CNS by playing multiple roles in regulating the etiopathology of MS, including alleviating demyelination, elevating the concentration of anti-inflammatory cytokines, and enhancing energy production (108). Multiple studies have also demonstrated the clinical relevance of the levels of estrogen to the risk and relapse rate of MS (109, 110). Functional studies have indicated that treatment with 17β-estradiol increases the remyelination of corpus axons and improves the signs and symptoms of animal models of MS (111–113). In this study, we found that the level of 17a-estradiol was significantly reduced in MS-affected individuals compared with that in healthy controls. This suggests that the decreased level of 17a-estradiol might participate in the pathophysiology and remission of MS, even though previous studies have consistently shown that 17β-estradiol rather than 17a-estradiol relieved associated symptoms in animal models of MS. However, the accurate effect of 17a-estradiol on the pathology of MS remains unclear, and its function needs to be determined in the future.

Furthermore, we observed that the level of spermidine was higher in the plasma of patients with MS than in healthy controls. An earlier study demonstrated that spermidine was able to alleviate the severity of murine EAE (114). Moreover, spermidine alleviation of EAE was shown to be mediated by the regulatory mechanism of infiltration of CD4^+^ T-cells and macrophages within the CNS (115). Also, spermidine is capable of conferring immunoregulatory potential in alleviating EAE symptoms through the indoleamine 2,3-dioxygenase 1 (IDO1) enzyme on dendritic cells (116). The upregulation of spermidine in the circulating system might contribute to the remission of MS in an undefined manner. Notably, the circulating levels of several types of fatty acids, such as saturated palmitic acid, polyunsaturated arachidonic acid, and monounsaturated oleic acid, were prominently increased in patients with MS compared with healthy controls. MS is a neuroinflammatory disease characterized by chronic demyelination. Lipids, specifically oleic acid, are important constituents of myelin. Early studies have indicated that the white matter in the CNS of patients with MS is composed mainly of oleic, stearic, and palmitic acid and reduced amounts of arachidonic acid (117). Previous studies suggested that the levels of palmitic and oleic acid were significantly increased in the plasma of MS-affected subjects compared with those in controls (118), consistent with our results. Specific lipids, such as oleic acid, could serve as potential biomarkers for demyelination (119). The accumulation of oleic, palmitic, and arachidonic acid in the circulating system might be due to chronic demyelination and the formation of white matter lesions. However, the biological relevance of these fatty acids in the course of MS remains to be verified. In addition to fatty acids, monoamine metabolites, such as homovanillic acid (HVA) and 5-hydroxyindoleacetic acid (5-HIAA), the respective principal metabolites of dopamine and serotonin, were significantly reduced in MS-affected patients compared to healthy subjects, consistent with the results of a previous study (120). The reduced levels of these two neurotransmitter metabolites, combined with other potential biomarkers, might be used to predict disease severity and movement disability in MS, even though the mechanisms through which these monoamine metabolites reversely regulate the progression of MS and the activities of neurotransmitters remain unclear. Interestingly, we also observed remarkable decreases in the level of dehydroepiandrosterone (DHEA), a neurosteroid, in patients with MS as compared to healthy controls. Neurosteroids function as key regulators of neuroinflammation in neurodegenerative diseases, including MS (121). Our data was consistent with a previous result of DHEA concentration was reduced in CNS tissues from patients with MS and in animals with EAE (122). Recent study also demonstrated that progressive resistance training, an exercise intervention, increased the abundances of dehydroepiandrosterone sulfate (DHEAS), and change in DHEAS levels reversely associated with the improvement of fatigue scores (123). Administration of either DHEA or DHEAS significantly reduced the severity, incidence, and neurobehavioral deficits of EAE (122, 124). Moreover, a recent study suggested that BNN20, a C17-spiroepoxy derivative of DHEA, could function as a neuroprotective molecule to reduce inflammation by binding to the neurotrophin receptor (125). The reduction in the levels of DHEA in patients with MS might play a role in facilitating the pathophysiology and progression of this condition.

Common and different characteristics have been found in metabolome studies of Chinese and Caucasian patients with MS. The most significant changed plasma metabolites in MS-affected Chinese patients were amino acid, lipid and their derivatives, of which representative differentially abundant metabolites such as L-glutamic acid, L-leucine and L-isoleucine, as well as sphingosine 1-phosphate and sphinganine 1-phosphate, were also identified in Caucasian populations with MS (26, 44, 107). Moreover, the increased levels of circulating fatty acids such as palmitic acid, arachidonic acid and oleic acid in Chinese patients with MS was also similar to findings of previous studies of Caucasian populations (117–119). The plasma concentrations of monoamine metabolites such as HVA and 5-HIAA, was reduced significantly in MS-affected Chinese patients, which was consistent with the results of previous study (109). However, some particular differential metabolites in Chinese patients with MS was significantly differs from that of MS-affected Caucasian populations. For instance, previous study suggested that the level of phenylalanine was prominently elevated in the CSF of Caucasian patients with MS (105), which contradicted our findings. Unlike the alteration of phenylalanine level, the abundances of arginine in both CSF and serum biospecimens showed no apparent differences between MS-affected Caucasian subjects and matched healthy controls (38), whereas, we found that the level of beta-alanyl-L- arginine increased dramatically in Chinese patients with MS compared to healthy subjects. Besides, the concentration of 17a-estradiol, rather than 17β-estradiol that was well-investigated in Caucasian populations and in animal models of MS (111–113, 126), was altered significantly in Chinese patients with MS. In addition, the plasma level of myo-inositol decreased greatly in Chinese patients with MS, which was inconsistent with the results from early study (127). Furthermore, unlike monoamine metabolites, polyamine like spermidine, was increased obviously in the circulating level of MS-affected Chinese patients. These contradictory findings of metabolomic studies in different populations could be attributed to several factors. For example, what type of biospecimens detected (plasma or CSF), and what types of populations investigated. The factors of ethnic background heterogeneity, as well as various lifestyle and dietary habits, cannot be ignored. Therefore, it brings us a huge challenge to identify *bona fide* universal biomarkers for MS.

In addition, we observed extensive alterations in the concentrations of pro- and anti-inflammatory cytokines and chemokines in the peripheral system of MS-affected individuals compared with those in healthy controls. The concentrations of IL-17 and TNF-α, two important proinflammatory cytokines, were prominently increased, whereas those of certain chemokines, such as RANTES, MIP-1α, MIP-1β, and MCP-1, were markedly decreased in MS-affected patients compared with those in healthy controls. Inflammation in the CNS of patients with MS or EAE is known to be primarily mediated by T helper type 1 (Th1) (67, 68) and Th17 T-cell (69–71) subsets that produce proinflammatory cytokines, such as TNF-α and IL-17, which have suppressive effects on the T regulatory (Treg) anti-inflammatory T-cell subset. In the current study, we observed that patients with MS displayed significantly increased levels of IL-17 and TNF-α and notably reduced concentrations of anti-inflammatory cytokines and chemokines, such as IL-7, MIP-1α, and MIP-1β, compared with healthy subjects. The extensive changes in the levels of these cytokines and chemokines are most likely due to the imbalance in the Th1/Th17 and Treg cell subsets in patients with MS. Consequently, the upregulation of IL-17 and TNF-α suppresses the functionality of Tregs and the release of inhibitory cytokines, such as IL-10, thus promoting the infiltration of inflammatory cells and inflammatory responses in the CNS. Similarly, a recent study reported that patients with MS exhibited significantly reduced numbers of Treg cells and an elevated percentage of Th17 cells compared with healthy controls (65). Supplementation with propionic acid restored the imbalance of Treg/Th17 cells (65). Moreover, another study showed that tryptophan metabolites and the IDO enzyme suppressed Th1 and Th17 cell differentiation in EAE (82). Administration of 3-hydroxyanthranilic acid (3-HAA), a downstream tryptophan metabolite, ameliorated the symptoms of EAE by increasing the number of Treg cells and inhibiting the functions of Th1/Th17 cells (82). The evident reduction in the level of L-tryptophan in Chinese patients with MS might be one of the causal factors enhancing the production of TNF-α and IL-17, thus reducing the production of inhibitory cytokines by Tregs, eventually accelerating the transition of inflammatory reactions from the peripheral system into the CNS.

However, this study had several limitations. First, the number of patients with MS and healthy controls was relatively small, which might have led to inaccurate findings. Hence, it is essential to perform further validation assays on an expanded sample group to consolidate the present findings. Second, only several specific cytokines and chemokines, rather than immune omics, were investigated, which might have provided incomplete information on immunological alterations. Third, our present study suggested only a tight correlation between changes in the levels of metabolites and that of cytokines and chemokines in MS-affected Chinese patients; the functional validation of these differentially abundant metabolites is still lacking. In the future, functional studies regarding certain metabolites should be conducted *in vitro* and in EAE models to provide stronger evidence that altered metabolic pathways coordinate the immune system to maintain the immunoinflammatory state of MS. This could facilitate a deeper understanding of the fundamentals of the metabolic-immune crosstalk-regulated pathogenesis of MS.

In conclusion, we dissected the altered metabolic profiles and immunoinflammatory responses of the peripheral system in Chinese patients with stable MS, revealing that amino acid and lipid metabolites might play potential roles in regulating pro-inflammatory responses in the circulatory system and in maintaining the course of MS. These findings might provide potential cues for developing therapeutic strategies for the management of MS in the future.

## Data Availability Statement

The original contributions presented in the study are included in the article/**Supplementary Material**. Further inquiries can be directed to the corresponding authors.

## Ethics Statement

The studies involving human participants were reviewed and approved by The Ethics Committee of the Second People’s Hospital of Lishui (Zhejiang, China). The patients/participants provided their written informed consent to participate in this study.

## Author Contributions

FY designed and performed the experiments, analyzed the data, and wrote the manuscript. S-CW designed the experiments, analyzed the data, and edited the manuscript. L-JZ and X-MY collected samples. Z-XL and S-CW analyzed the data. LH, L-MY, and L-YZ supervised the study and edited the manuscript. All authors contributed to the article and approved the submitted version.

## Funding

This study was funded by grants from the S&T Major Project of Lishui City (2017ZDYF15) and the Research Fund for Lin He Academician New Medicine.

## Conflict of Interest

The authors declare that the research was conducted in the absence of any commercial or financial relationships that could be construed as a potential conflict of interest.

## Publisher’s Note

All claims expressed in this article are solely those of the authors and do not necessarily represent those of their affiliated organizations, or those of the publisher, the editors and the reviewers. Any product that may be evaluated in this article, or claim that may be made by its manufacturer, is not guaranteed or endorsed by the publisher.

## Abbreviations

3-HAA, 3-hydroxyanthranilic acid; 5-HIAA, 5-hydroxyindoleacetic acid; AHR, aryl hydrocarbon receptor; CIS, clinically isolated syndrome; CNS, central nervous system; CSF, cerebrospinal fluid; DAM, differentially abundant metabolite; DHEA, dehydroepiandrosterone; DHEAS, dehydroepiandrosterone sulfate; EAE, experimental autoimmune encephalomyelitis; HRMS, high-resolution mass spectrometry; HVA, homovanillic acid; IDO1, indoleamine 2,3-dioxygenase 1; MS, multiple sclerosis; MSIF, multiple sclerosis international federation; OPLS-DA, orthogonal partial least squares discriminant analysis; PCA, principal component analysis; PLS-DA, partial least squares-discriminant analysis; PPMS, primary progressive multiple sclerosis; RRMS, relapsing-remitting multiple sclerosis; RSD, relative standard deviation; SCFAs, short-chain fatty acids; UPLC, ultra-performance liquid chromatography; VIP, variable importance for the projection.
