# Supplementary material for: Altered Plasma Metabolic Profiles in Chinese Patients With Multiple Sclerosis
Source: Front Immunol. 2021 Dec 15;12:792711. doi: 10.3389/fimmu.2021.792711 (PMC8715987; doi:10.3389/fimmu.2021.792711)

# Supplementary Material

**Table S1**. **VIP scores of metabolites driving separation between patients with MS and healthy controls (VIP ≥ 1.000, *P* value < 0.05)**

| **Metabolites** | **VIP value** |
| --- | --- |
| myo-inositol | 2.430 |
| sphingosine 1-phosphate | 2.417 |
| 17a-estradiol | 2.412 |
| L-tyrosine | 2.345 |
| uridine | 2.160 |
| methyl jasmonate | 2.152 |
| L-isoleucine | 2.096 |
| sphinganine 1-phosphate | 2.051 |
| L-glutamic acid | 1.894 |
| trans-cinnamate | 1.878 |
| O-phosphoethanolamine | 1.784 |
| L-arogenate | 1.751 |
| 4-oxoglutaramate | 1.748 |
| pseudouridine | 1.744 |
| N-acetyl-L-aspartic acid | 1.700 |
| dehydroepiandrosterone | 1.670 |
| cis-4-hydroxy-D-proline | 1.666 |
| L-leucine | 1.654 |
| deoxyuridine | 1.648 |
| sorbitol | 1.635 |
| L-tryptophan | 1.593 |
| palmitic acid | 1.583 |
| phytosphingosine | 1.572 |
| beta-alanyl-L-arginine | 1.552 |
| spermidine | 1.542 |
| 2-dehydropantoate | 1.529 |
| creatinine | 1.482 |
| arachidonic acid | 1.362 |
| niacinamide | 1.344 |
| L-phenylalanine | 1.340 |
| homovanillic acid | 1.320 |
| myristic acid | 1.319 |
| nicotinuric acid | 1.269 |
| dodecanoic acid | 1.268 |
| oleic acid | 1.229 |
| 5-hydroxyindoleacetic acid | 1.211 |
| isocitric acid | 1.189 |
| L-valine | 1.149 |
| acetylphosphate | 1.144 |
| alpha-dimorphecolic acid | 1.124 |
| skatole | 1.011 |
| betaine | 1.005 |

**Table S2**. **Intensity of metabolites significantly altered between patients with MS and healthy controls**

| **Metabolites** | **MS (mean ± SD)** | **Control (mean ± SD)** | **Fold change (MS/Control)** | ***P* value** | ***Padj* value (BH correction)** |
| --- | --- | --- | --- | --- | --- |
| beta-alanyl-L-arginine | 3.01 ± 2.80 | 0.81 ± 0.67 | 3.70 | 8.35e-04 | 5.61e-03 |
| methyl jasmonate | 17.25 ± 9.08 | 5.56 ± 2.47 | 3.10 | 6.58e-06 | 1.63e-04 |
| 4-oxoglutaramate | 1.51 ± 1.06 | 0.50 ± 0.37 | 3.00 | 7.01e-04 | 4.90e-03 |
| isocitric acid | 0.73 ± 0.71 | 0.30 ± 0.35 | 2.43 | 4.31e-03 | 1.87e-02 |
| L-glutamic acid | 4.27 ± 2.25 | 1.92 ± 0.73 | 2.22 | 5.22e-06 | 3.27e-04 |
| L-valine | 0.66 ± 0.50 | 0.38 ± 0.18 | 1.75 | 1.56e-02 | 5.04e-02 |
| O-phosphoethanolamine | 0.66 ± 0.24 | 0.38 ± 0.18 | 1.72 | 3.39e-04 | 2.84e-03 |
| sorbitol | 1.33 ± 0.61 | 0.78 ± 0.27 | 1.70 | 1.65e-03 | 9.30e-03 |
| spermidine | 0.88 ± 0.41 | 0.53 ± 0.19 | 1.64 | 2.49e-03 | 1.25e-02 |
| oleic acid | 2.09 ± 0.67 | 1.71 ± 0.12 | 1.23 | 1.11e-02 | 4.58e-02 |
| palmitic acid | 1.22 ± 0.16 | 1.07 ± 0.11 | 1.14 | 9.12e-04 | 8.05e-03 |
| arachidonic acid | 1.08 ± 0.04 | 1.06 ± 0.01 | 1.02 | 2.29e-03 | 1.50e-02 |
| L-phenylalanine | 1.44 ± 0.20 | 1.61 ± 0.19 | 0.89 | 9.01e-03 | 3.30e-02 |
| dehydroepiandrosterone | 0.96 ± 0.11 | 1.08 ± 0.09 | 0.89 | 1.52e-03 | 1.14e-02 |
| 2-dehydropantoate | 1.19 ± 0.22 | 1.40 ± 0.18 | 0.85 | 2.12e-03 | 1.11e-02 |
| skatole | 0.94 ± 0.33 | 1.12 ± 0.24 | 0.83 | 3.78e-02 | 9.95e-02 |
| 5-hydroxyindoleacetic acid | 0.86 ± 0.19 | 1.03 ± 0.26 | 0.83 | 3.35e-02 | 9.07e-02 |
| cis-4-hydroxy-D-proline | 1.61 ± 0.46 | 2.07 ± 0.31 | 0.78 | 3.39e-04 | 4.10e-03 |
| L-tryptophan | 1.09 ± 0.46 | 1.54 ± 0.35 | 0.71 | 2.29e-03 | 1.50e-02 |
| myristic acid | 1.76 ± 0.47 | 2.48 ± 1.09 | 0.71 | 1.46e-02 | 4.81e-02 |
| N-acetyl-L-aspartic acid | 1.60 ± 0.43 | 2.27 ± 0.68 | 0.70 | 7.65e-04 | 7.24e-03 |
| homovanillic acid | 1.20 ± 0.56 | 1.74 ± 0.67 | 0.69 | 5.82e-03 | 2.34e-02 |
| L-tyrosine | 0.90 ± 0.17 | 1.36 ± 0.27 | 0.66 | 8.65e-07 | 5.95e-05 |
| creatinine | 1.16 ± 0.52 | 1.75 ± 0.65 | 0.66 | 1.95e-03 | 1.05e-02 |
| nicotinuric acid | 0.76 ± 0.35 | 1.16 ± 0.57 | 0.66 | 6.27e-03 | 2.49e-02 |
| trans-cinnamate | 1.47 ± 0.52 | 2.25 ± 0.60 | 0.66 | 2.56e-04 | 3.32e-03 |
| deoxyuridine | 1.53 ± 0.49 | 2.43 ± 1.00 | 0.63 | 9.12e-04 | 5.97e-03 |
| L-arogenate | 0.87 ± 0.31 | 1.43 ± 0.57 | 0.60 | 1.80e-03 | 1.27e-02 |
| betaine | 0.54 ± 0.54 | 0.94 ± 0.72 | 0.57 | 3.35e-02 | 9.07e-02 |
| pseudouridine | 0.71 ± 0.25 | 1.26 ± 0.59 | 0.56 | 3.09e-04 | 3.79e-03 |
| sphingosine 1-phosphate | 1.00 ± 0.46 | 1.87 ± 0.31 | 0.54 | 1.60e-06 | 7.41e-05 |
| myo-inositol | 0.64 ± 0.23 | 1.25 ± 0.31 | 0.52 | 9.79e-07 | 6.15e-05 |
| sphinganine 1-phosphate | 0.94 ± 0.58 | 1.87 ± 0.58 | 0.50 | 3.09e-05 | 5.10e-04 |
| L-leucine | 1.51 ± 1.00 | 3.04 ± 1.58 | 0.50 | 7.01e-04 | 4.90e-03 |
| phytosphingosine | 1.38 ± 1.41 | 2.86 ± 1.33 | 0.48 | 1.52e-03 | 8.71e-03 |
| acetylphosphate | 1.04 ± 0.58 | 2.18 ± 2.13 | 0.48 | 2.03e-02 | 6.16e-02 |
| 17a-estradiol | 0.50 ± 0.22 | 1.11 ± 0.34 | 0.45 | 3.27e-06 | 2.65e-04 |
| uridine | 1.57 ± 0.78 | 3.55 ± 1.43 | 0.44 | 4.14e-06 | 2.86e-04 |
| dodecanoic acid | 0.40 ± 0.20 | 0.95 ± 0.93 | 0.42 | 3.16e-03 | 1.51e-02 |
| niacinamide | 1.85 ± 1.86 | 5.74 ± 5.96 | 0.32 | 1.11e-02 | 3.87e-02 |
| L-isoleucine | 1.71 ± 1.16 | 12.86 ± 9.54 | 0.13 | 2.91e-06 | 9.50e-05 |
| alpha-dimorphecolic acid | 10.17 ± 13.15 | 167.37 ± 310.85 | 0.06 | 1.29e-03 | 7.64e-03 |

**Table S3**. **Concentrations of cytokines and chemokines significantly changed between patients with MS and healthy controls**

| **Cytokines/ Chemokines** | **MS (mean ± SD)** | **Control (mean ± SD)** | **Fold change (MS/Control)** | ***P* value** | ***Padj* value (BH correction)** |
| --- | --- | --- | --- | --- | --- |
| TNF-α | 142.94 ± 72.61 | 62.46 ± 30.82 | 2.29 | 1.13e-04 | 7.57e-04 |
| IL-17 | 20.96 ± 6.42 | 12.17 ± 7.00 | 1.72 | 2.37e-04 | 7.57e-04 |
| IL-9 | 166.66 ± 59.84 | 206.82 ± 27.46 | 0.81 | 1.14e-02 | 1.82e-02 |
| MIP-1β | 255.32 ± 83.49 | 351.00 ± 57.22 | 0.73 | 1.88e-04 | 7.57e-04 |
| RANTES | 8587.37 ± 5348.83 | 13050.11 ± 3085.55 | 0.66 | 3.10e-03 | 5.51e-03 |
| PDGF-bb | 1493.16 ± 1161.74 | 2712.39 ± 851.98 | 0.55 | 6.42e-04 | 1.67e-03 |
| IL-1ra | 138.34 ± 116.02 | 256.86 ± 166.31 | 0.54 | 1.51e-02 | 2.01e-02 |
| MCP-1 | 29.16 ± 20.18 | 59.01 ± 24.29 | 0.49 | 1.94e-04 | 7.57e-04 |
| IL-7 | 5.51 ± 4.66 | 12.82 ± 10.90 | 0.43 | 1.25e-02 | 1.82e-02 |
| IL-12 | 1.18 ± 1.03 | 3.34 ± 2.26 | 0.35 | 8.28e-04 | 1.67e-03 |
| MIP-1α | 1.72 ± 1.77 | 19.64 ± 19.49 | 0.09 | 8.36e-04 | 1.67e-03 |
| IL-8 | 5.17 ± 4.36 | 68.40 ± 54.13 | 0.08 | 7.59e-05 | 7.57e-04 |
| IL-13 | 11.74 ± 10.29 | 13.45 ± 13.18 | 0.87 | 0.65 | 0.65 |
| Eotaxin | 27.14 ± 21.29 | 31.55 ± 10.01 | 0.86 | 0.41 | 0.44 |
| IP-10 | 274.32 ± 191.90 | 362.29 ± 163.45 | 0.76 | 0.13 | 0.15 |
| IFN-γ | 4.40 ± 5.75 | 6.92 ± 2.91 | 0.64 | 0.09 | 0.11 |

**Supplementary Figure Legends**

**Fig. S1 PLS-DA and PCA models for separating MS-affected patients and healthy controls.** (**A**) and (**B**) PLS-DA plots for the positive ion model. Pre (principal component score) = 3, the respective model interpretability for X and Y variable dataset was R2X = 0.307 and R2Y = 0.989, model predictability Q2 = 0.902. (**C**) and (**D**) PLS-DA plots for the negative ion model. Pre (principal component score) = 3, the respective model interpretability for X and Y variable dataset was R2X = 0.216 and R2Y = 0.993, model predictability Q2 = 0.851. (**E**) PCA plot for the positive ion model. Pre (principal component score) = 8, for X variable dataset, model interpretability R2X = 0.509. (**F**) PCA plot for the negative ion model. Pre (principal component score) = 10, for X variable dataset, model interpretability R2X = 0.502.

**Fig. S2 Classification of all identified metabolites.** The pie chart was plotted using the KEGG and Metabolon databases. The majority of all identified metabolites was classified into the following categories: "amino acid" (33.0%), "lipid" (28.0%), "cofactors and vitamins" (11.0%), "nucleotide" (10.0%), "carbohydrate" (9.0%), and "xenobiotics" (8.0%).

**Fig. S3 Significantly up- and downregulated metabolites in patients with MS.** (**A**) Z-score plot of all 42 DAMs. The Z-score plot was standardized on the original data, reflecting the distribution of the relative content of each differential metabolite in different samples. The formula used for this calculation was: z = (x-μ)/σ, x denotes a specific score, μ represents the average, and σ indicates the standard deviation. Each dot in the plot represents the relative amount of a certain metabolite in a single sample. (**B**) Normalized intensity of 30 DAMs in the plasma samples of 22 MS-affected patients and 21 healthy controls. Samples were compared using the T-test. *P* value was corrected using Benjamini-Hochberg procedure. *: *Padj* < 0.05, **: *Padj* < 0.01, ***: *Padj* < 0.001.

**Fig. S4 Correlation and pathway analysis of DAMs.** (**A**) Correlation chord diagram representing the overall correlation between all DAMs; each chord line indicates the correlation between a single metabolite and another metabolite; red and blue denote positive and negative correlations, respectively. (**B**) Correlation network of all DAMs. (**C**) Bar graph of enriched pathways of all DAMs in patients with MS.

**Fig. S5 Correlation between non-differential metabolites and the levels of cytokines and chemokines in patients with MS.** The heatmap was plotted using Pearson’s correlation analysis. The correlation coefficient is denoted by red and blue, representing positive and negative correlations, respectively. Asterisks indicate significant positive or negative correlations. *P* value was corrected using Benjamini-Hochberg procedure. *: *Padj* < 0.05.

**Fig. S6 Several differential metabolites were not significantly correlated with the level of cytokines and chemokines in MS-affected patients.** The heatmap was plotted using Pearson’s correlation analysis. The correlation coefficient is represented by red and blue that denote positive and negative correlations, respectively.

**Fig. S1**


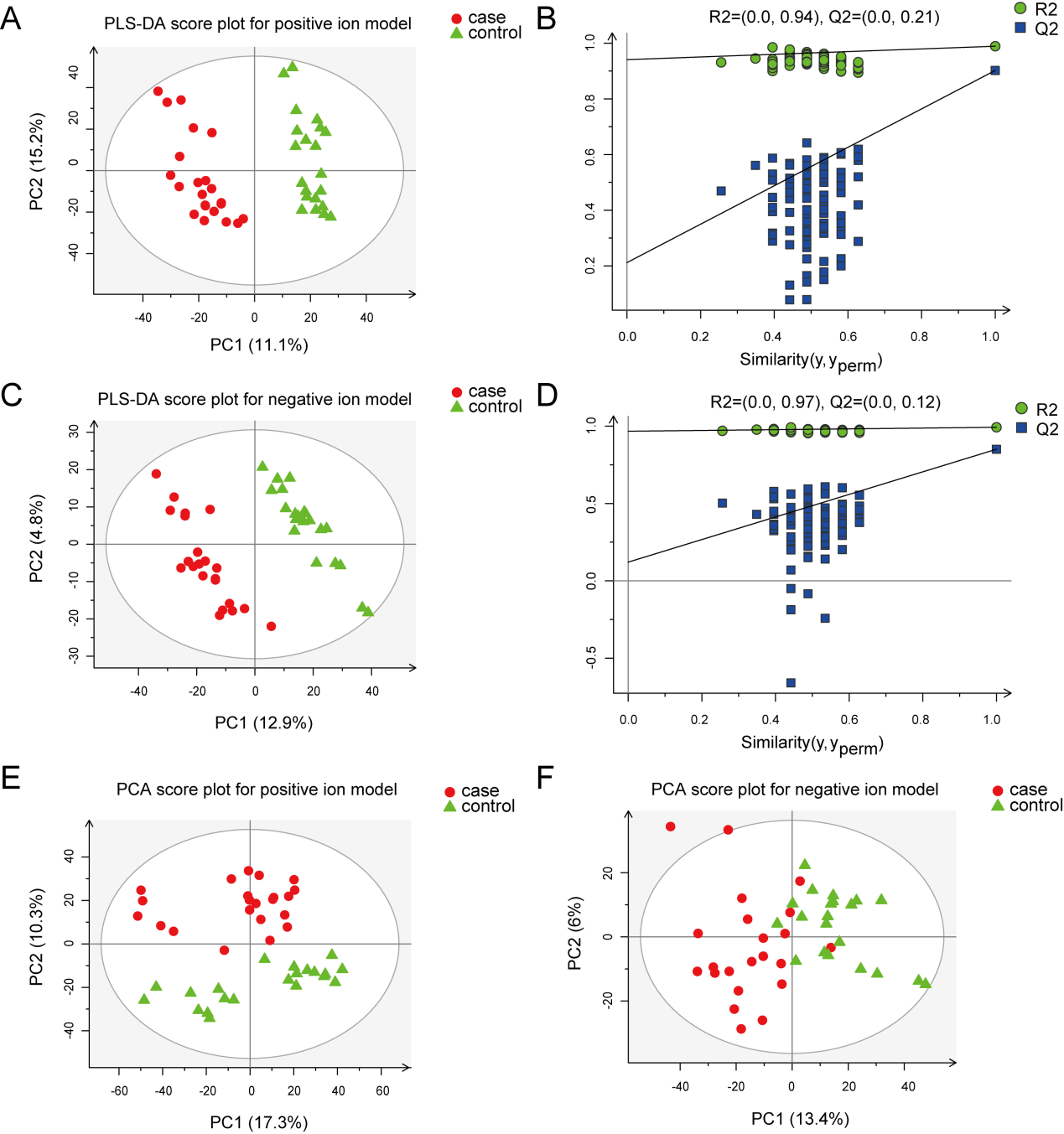


**Fig. S2**


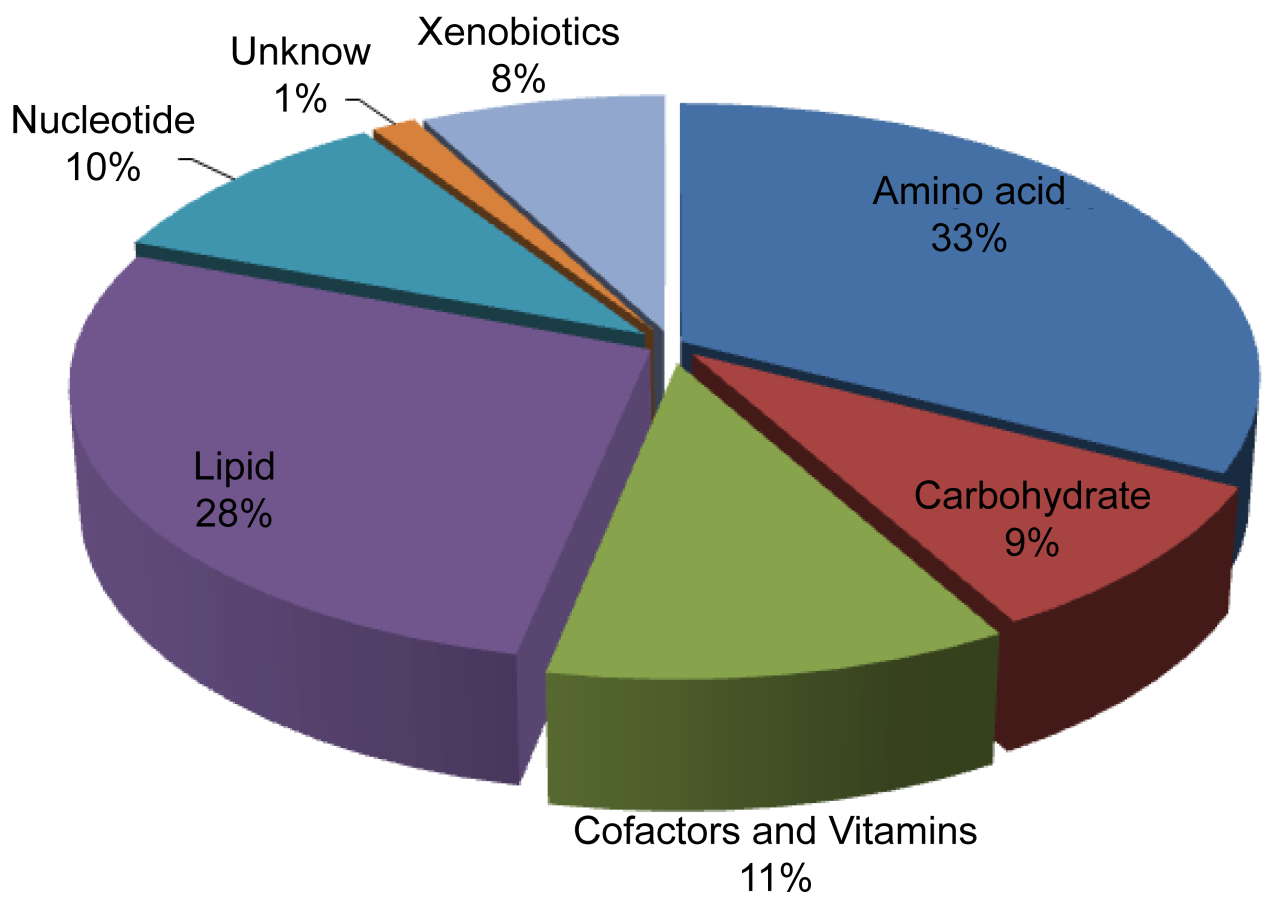


**Fig. S3**


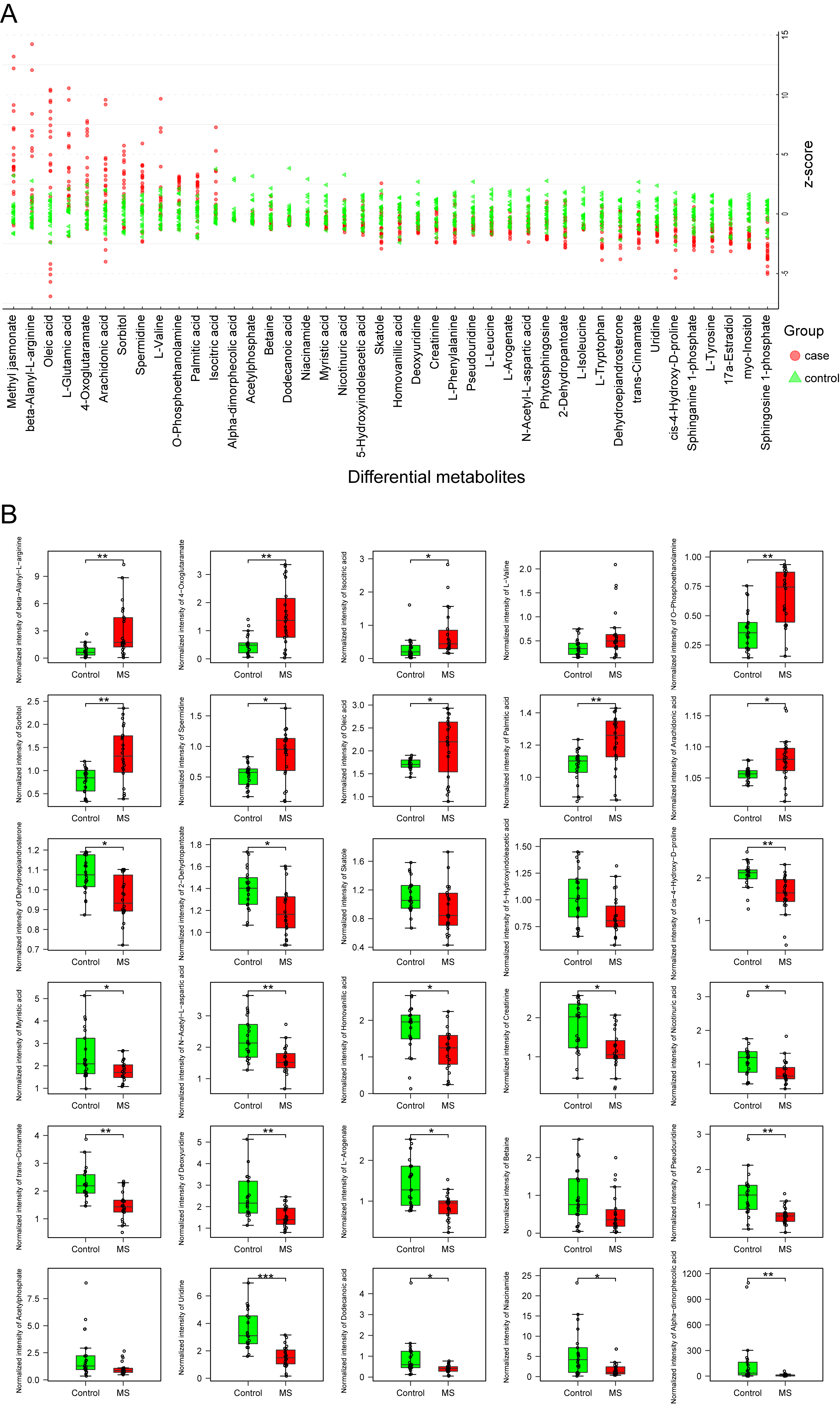


**Fig. S4**


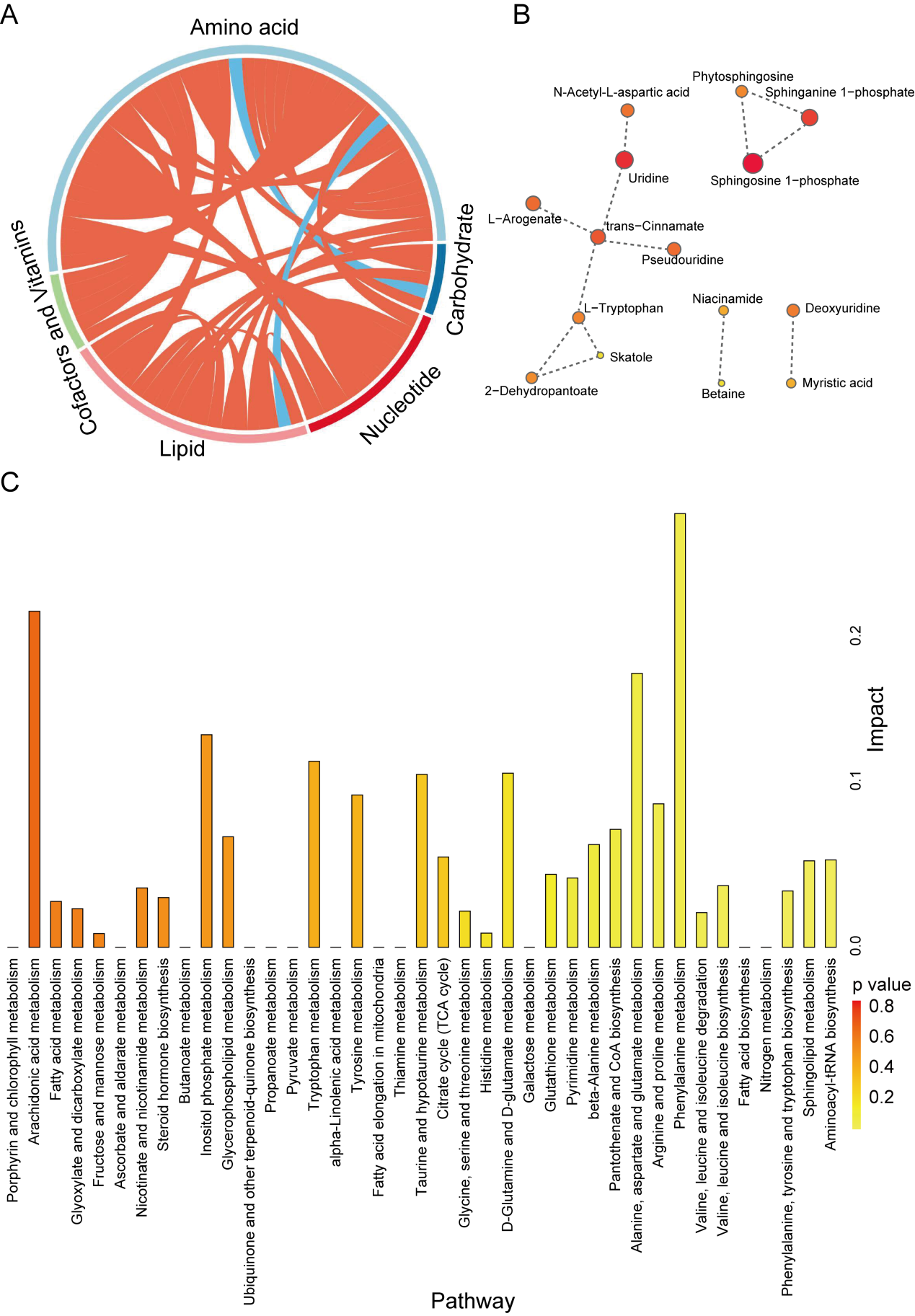


**Fig. S5**


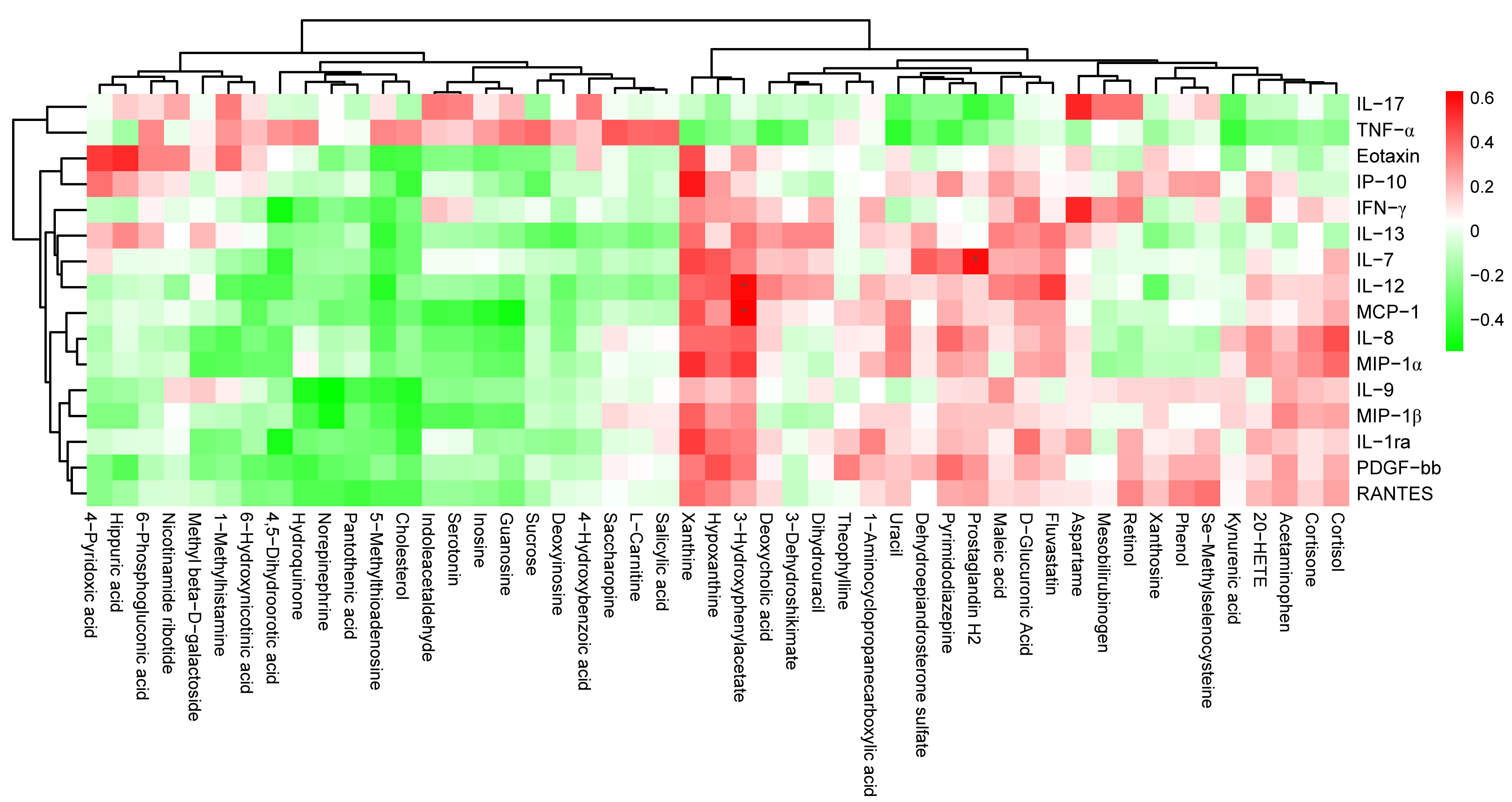


**Fig. S6**


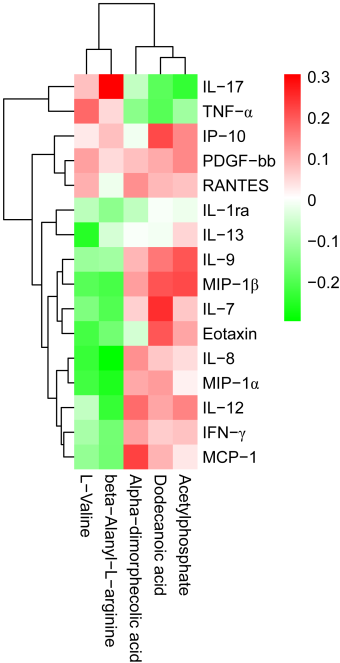

Supplement: Supplementary file 1 [file DataSheet_1.docx]
